# Supplementary material for: Estimation of free-roaming domestic dog population size: Investigation of three methods including an Unmanned Aerial Vehicle (UAV) based approach
Source: PLoS One. 2020 Apr 8;15(4):e0225022. doi: 10.1371/journal.pone.0225022 (PMC7141685; doi:10.1371/journal.pone.0225022)
Supplement: S1 Fig — A. La Romana, B. Sabaneta, C. Poptún study area. The green and the red dots are the starting and ending points, respectively. (PDF) [file pone.0225022.s001.pdf]

S1 Figure. Transects lines in the three study areas, Petén, Guatemala. A. La Romana, B. Sabaneta, C. Poptún study area. The green and the red dots are the starting and ending points, respectively. Source: USGS LandsatLook

A.

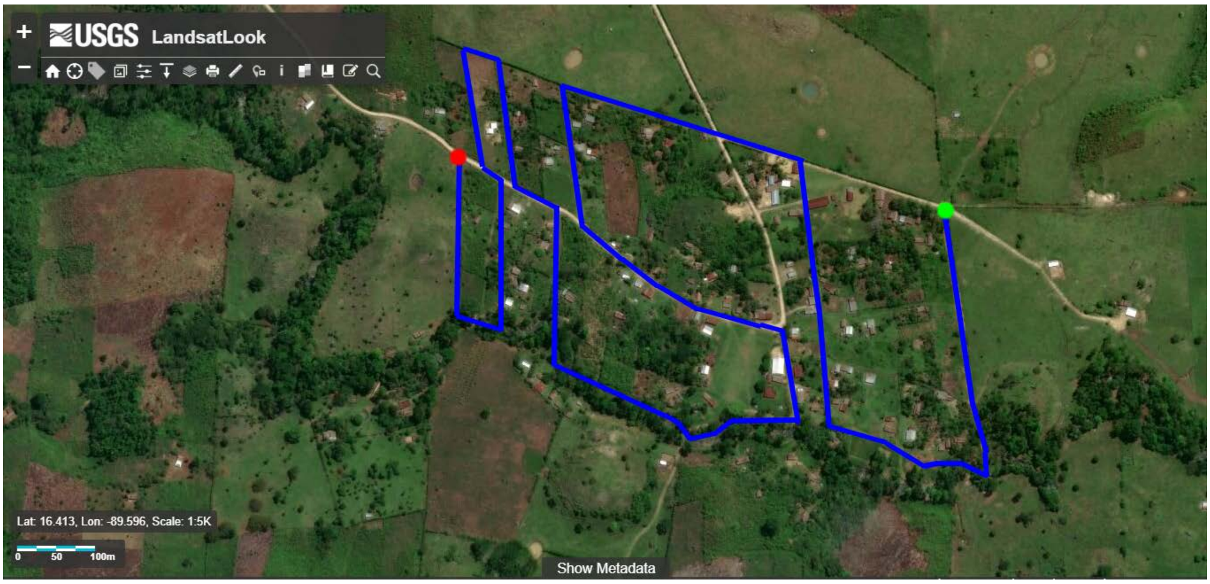

B.

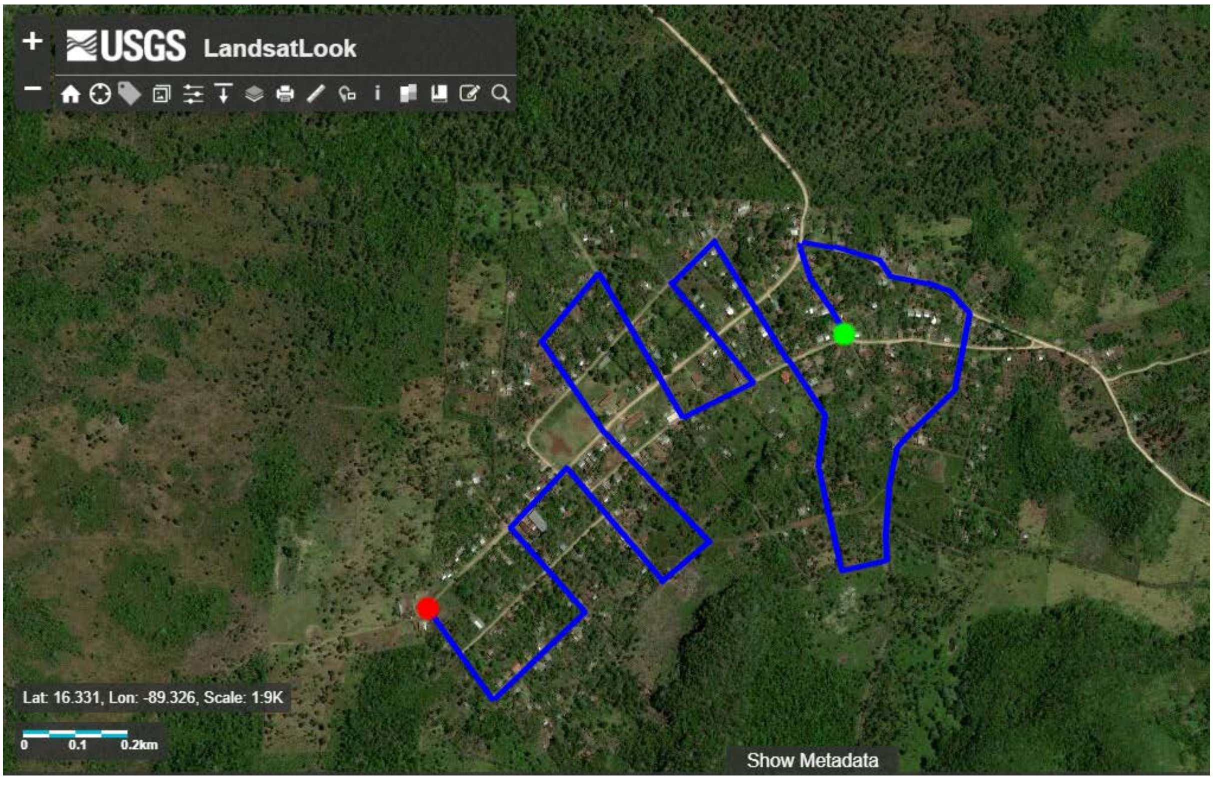

C.

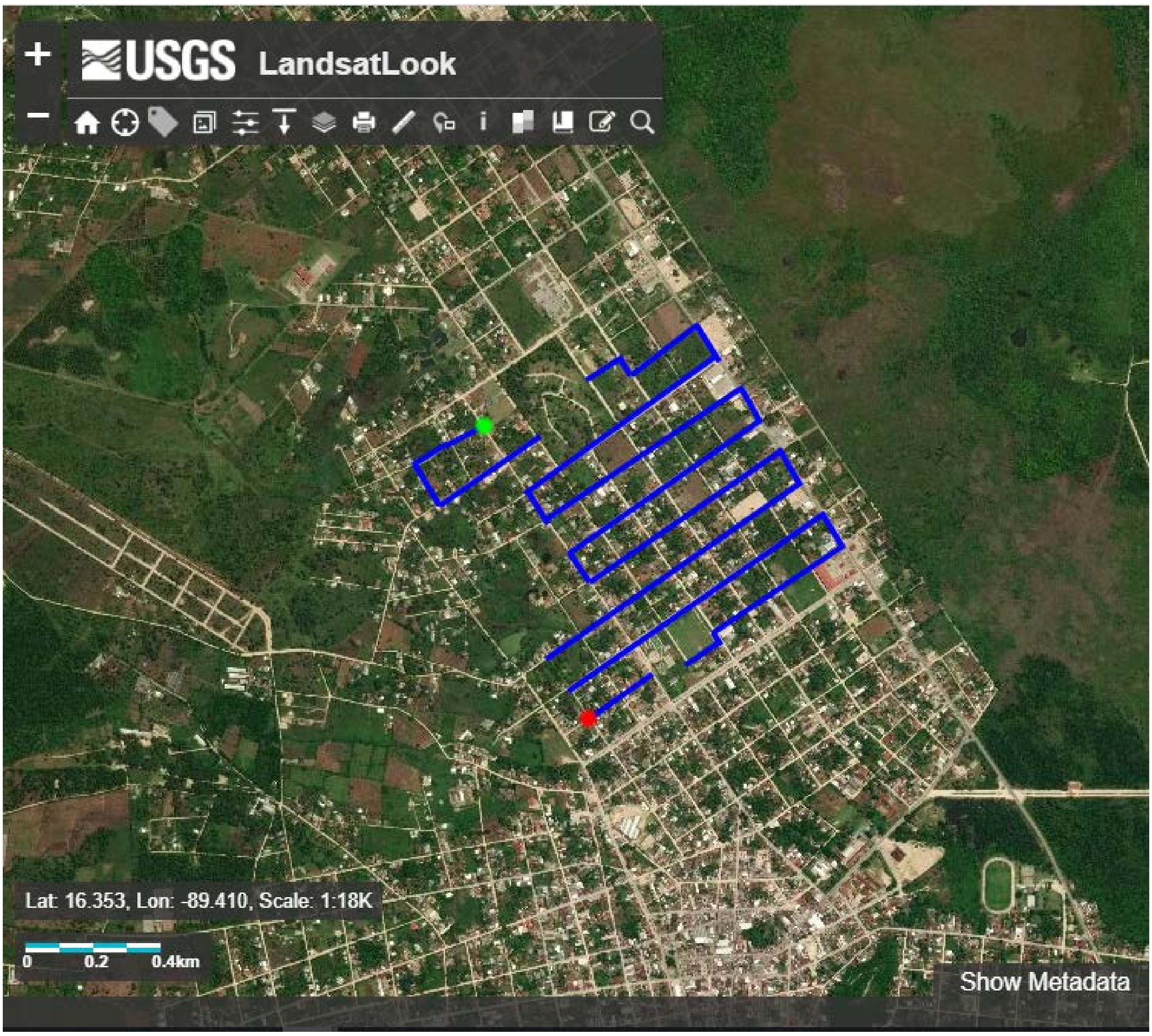
USGS LandsatLook
Lat: 16.353, Lon: -89.410, Scale: 1:18K
0 0.2 0.4km
Show Metadata
