# Supplementary figures and images for: Estimation of free-roaming domestic dog population size: Investigation of three methods including an Unmanned Aerial Vehicle (UAV) based approach
Source: PLoS One. 2020 Apr 8;15(4):e0225022. doi: 10.1371/journal.pone.0225022 (PMC7141685; doi:10.1371/journal.pone.0225022)

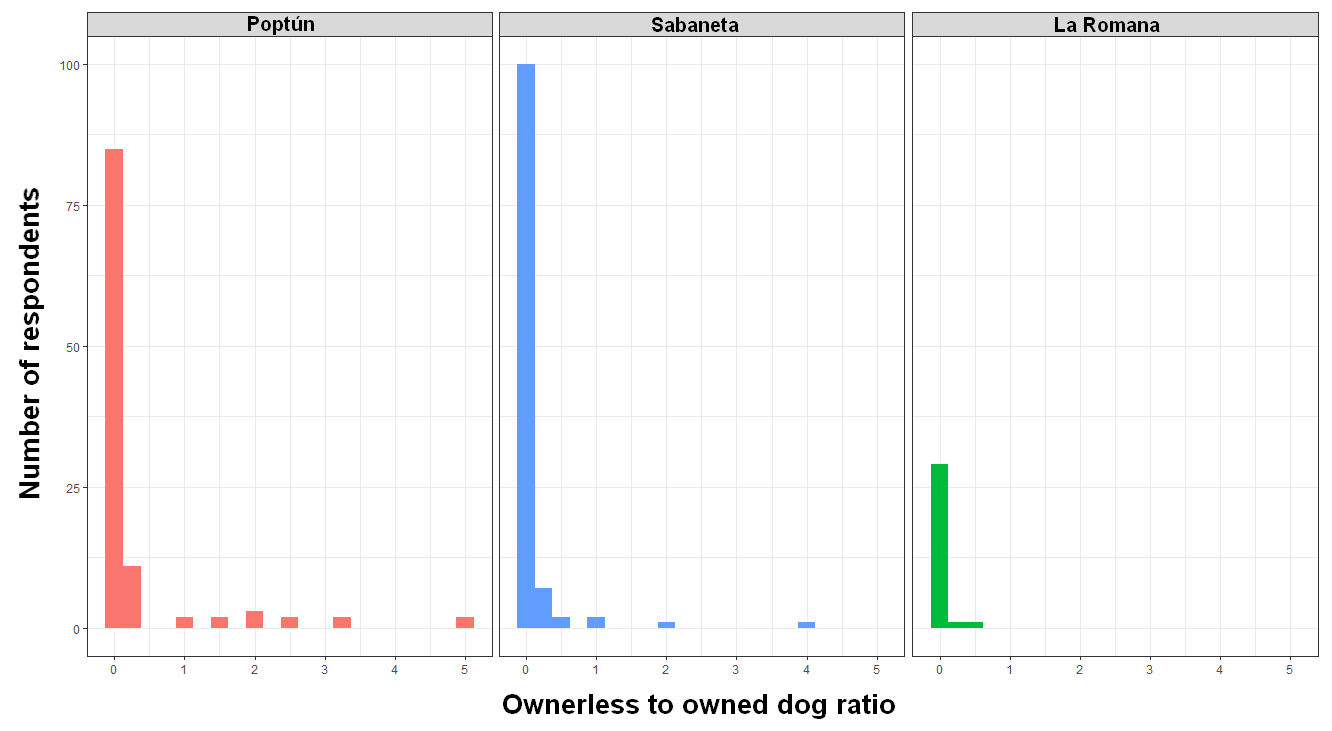

Supplement: S2 Fig — (PNG) [file pone.0225022.s002.png]
