# Supplementary material for: Estimation of free-roaming domestic dog population size: Investigation of three methods including an Unmanned Aerial Vehicle (UAV) based approach
Source: PLoS One. 2020 Apr 8;15(4):e0225022. doi: 10.1371/journal.pone.0225022 (PMC7141685; doi:10.1371/journal.pone.0225022)
Supplement: S1 Table — (PDF) [file pone.0225022.s003.pdf]

Community	Corner	Longitude	Latitude
La Romana	North-west	-89.595	16.414
	North-east	-89.586	16.414
	South-west	-89.595	16.405
	South-east	-89.586	16.405
Sabaneta	North-west	-89.328	16.329
	North-east	-89.319	16.329
	South-west	-89.328	16.320
	South-east	-89.319	16.320
Poptún study area	North-west	-89.432	16.345
	North-east	-89.422	16.337
	South-west	-89.425	16.337
	South-east	-89.416	16.342
