# Supplementary material for: Estimation of free-roaming domestic dog population size: Investigation of three methods including an Unmanned Aerial Vehicle (UAV) based approach
Source: PLoS One. 2020 Apr 8;15(4):e0225022. doi: 10.1371/journal.pone.0225022 (PMC7141685; doi:10.1371/journal.pone.0225022)
Supplement: S2 Table — The interviewees were dog owners whose household is located in the three study sites. (PDF) [file pone.0225022.s004.pdf]

Type of question	Question	Condition (if applicable)	Choices (if applicable)	Repeated question
**General information**				
Text	Questionnaire ID / Código de identificación del cuestionario	-	-	No
Date	Date / Fecha	-	-	No
Text	Name of the interviewee / Nombre del entrevistado	-	-	No
Text	Phone number / Número de teléfono	-	-	No
**Dog confinement practices**				
Text	Name of the dog / Nombre del perro	-	-	For each dog owned by the interviewee
Single choice	Can your dog have access to the streets without supervision? / ¿El perro	-	Yes / Sí<br>No / No	For each dog owned by the interviewee
Single choice	When? / ¿Cuando?	If "Yes" was selected in the previous question	All the time / Todo el tiempo<br>All day / Todo el dia<br>All night / Toda la noche<br>A few hours per day / Pocas horas por dia<br>Other (specify) / Otro<br>Do not know / No sé	For each dog owned by the interviewee
Text	Specify when / Especifica cuando	If "Other" was selected in the previous question	-	No
**Proportion of dogs based on this ownership status**				
Multiple choice	Which category of dog can we find in the town/village? / ¿Qué categorías de perros podemos encontrar en el pueblo?		Owned dogs / Perros con dueño<br>Ownerless dogs / Perros sin dueño<br>Community dogs / Perros de la comunidad<br>Other / Otro	No
Text	Specify the other category / Especifica la otra categoría	If "Other" was selected in the previous question	-	No
Integer	How many owned dogs live in the village? / ¿Cuántos perros con dueño cree usted que viven en el pueblo?	If "Owned dogs" was selected	-	No

Integer	How many ownerless dogs live in the village? / ¿Cuántos perros sin dueño cree usted que viven en el pueblo?	If "Ownerless dogs" was selected	-	No
Integer	How many community dogs live in the village? / ¿Cuántos perros de la comunidad cree usted que viven en el pueblo?	If "Community dogs" was selected	-	No
Integer	How many other dogs live in the village? ¿Cuántos otros perros cree usted que viven en el pueblo?	If "Other" was selected	-	No
