# Supplementary material for: Estimation of free-roaming domestic dog population size: Investigation of three methods including an Unmanned Aerial Vehicle (UAV) based approach
Source: PLoS One. 2020 Apr 8;15(4):e0225022. doi: 10.1371/journal.pone.0225022 (PMC7141685; doi:10.1371/journal.pone.0225022)
Supplement: S5 Table — (PDF) [file pone.0225022.s007.pdf]

	Number of dogs		
	La Romana	Sabaneta	Poptun
Always free-roaming	49	98	66
Free-roaming during the day only	2	0	4
Free-roaming during the night only	0	0	8
Free-roaming a few hours per day	2	13	20
Never free-roaming	7	13	18
