# Supplementary material for: Estimation of free-roaming domestic dog population size: Investigation of three methods including an Unmanned Aerial Vehicle (UAV) based approach
Source: PLoS One. 2020 Apr 8;15(4):e0225022. doi: 10.1371/journal.pone.0225022 (PMC7141685; doi:10.1371/journal.pone.0225022)
Supplement: S6 Table — (PDF) [file pone.0225022.s008.pdf]

		Number of marked dogs recaptured	Number of unmarked dogs recaptured
La Romana			
	1st transect	22	4
	2nd transect	21	8
	3rd transect	21	12
	4th transect	20	4
Sabaneta			
	1st transect	42	52
	2nd transect	38	42
	3rd transect	42	46
	4th transect	48	44
Poptún			
	1st transect	13	42
	2nd transect	24	45
	3rd transect	10	41
	4th transect	12	24
