# Supplementary material for: Estimation of free-roaming domestic dog population size: Investigation of three methods including an Unmanned Aerial Vehicle (UAV) based approach
Source: PLoS One. 2020 Apr 8;15(4):e0225022. doi: 10.1371/journal.pone.0225022 (PMC7141685; doi:10.1371/journal.pone.0225022)
Supplement: S7 Table — (PDF) [file pone.0225022.s009.pdf]

	La Romana	Sabaneta	Poptún
Gamma distribution parameters			
a	0.138943	0.1205953	0.1208189
b	0.3699105	0.3768178	0.3667614
Outcome			
Mean number of owned FRDD	75	239	352
(credibility interval)	(63-82)	(142-273)	(155-471)
Mean number of ownerless dogs	2	16	36
(credibility interval)	(0-12)	(0-98)	(0-161)
Mean total number of FRDD	77	256	389
(credibility interval)	(74-82)	(236-274)	(296-392)
