# Supplementary material for: Estimation of free-roaming domestic dog population size: Investigation of three methods including an Unmanned Aerial Vehicle (UAV) based approach
Source: PLoS One. 2020 Apr 8;15(4):e0225022. doi: 10.1371/journal.pone.0225022 (PMC7141685; doi:10.1371/journal.pone.0225022)
Supplement: S1 File — (PDF) [file pone.0225022.s010.pdf]

S1 File. Script of the Bayesian statistical model implemented on OpenBUGS.

Model

```
model {

for (i in 1:zone) {
        for( t in 1 : T ) {

                        x[i,t] ~ dbin(p[i,t],n1[i,t])
                        z[i,t] ~ dbin(p[i,t],n2[i,t])


                        c[i,t] ~ dunif(cmin[i],cmax[i])

                        p[i,t]~dunif(pmin[i],pmax[i])

                        lm[i,t] <-(Ml[i]/Mc[i])*(t/4)

                n1[i,t]<-round(p[i,t]*Mc[i]*(1-lm[i,t])*(1-c[i,t]))
                n2[i,t]<-round(p[i,t]*((1-c[i,t])*(M[i]-Mc[i] + lm[i,t]*Mc[i]) + aa[i]*M[i]))

                        }

                M[i] ~ dunif(Mmin[i],Mmax[i])

                aa[i] ~ dlnorm(mu[i],tau[i])
                N[i] <- round(M[i] + aa[i]*M[i])

                Mol[i] <- aa[i]*M[i]
        }
}
```

Data

zone: number of zones
T: number of of transects per zone
x: number of captured marked owned dogs
z : number of captured unmarked dogs (owned + ownerless)
c: confinement probability
cmin/cmax: prior parameters of a uniform distribution for the confinement probability
P: recapture probability (coverage*encountering*reporting)
pmin/pmax : prior parameters of a Uniform distribution for the recapture probabilities
lm: probability of losing a collar
Ml : total number of lost collars
N : total number of free-roaming dogs (owned and ownerless)
Mc : total number of marked dogs
M : total number of owned dogs (marked and non-marked)
Mmin/Mmax: prior parameters of a uniform distribution for the total number of owned dogs
aa: ownerless to owned dogs ratio

```
list(T=4, zone=3,
x=structure(.Data=c(22,21,21,20,42,38,42,48,13,24,10,12),.Dim=c(3,4)),
z=structure(.Data=c(4,6,10,2,52,42,46,44,42,45,41,24),.Dim=c(3,4)),
Mc=c(61,125,118),
Ml=c(3,2,4),
Mmin=c(61,125,118), Mmax=c(1000,1000,1000),
pmin=c(0.28,0.14,0.17), pmax=c(0.89,0.71,0.68),
cmin=c(0.133,0.157,0.293), cmax=c(0.142,0.157,0.476),
mu=c(-6.573127,-7.713660,-7.667142),tau=c(5.237232,4.970247,4.984291))
```
