# Supplementary material for: Estimation of free-roaming domestic dog population size: Investigation of three methods including an Unmanned Aerial Vehicle (UAV) based approach
Source: PLoS One. 2020 Apr 8;15(4):e0225022. doi: 10.1371/journal.pone.0225022 (PMC7141685; doi:10.1371/journal.pone.0225022)
Supplement: Attachment — Submitted filename: Response to Reviewers.docx [file pone.0225022.s011.docx]

# Response to the journal requirements:

1. When submitting your revision, we need you to address these additional requirements.
Please ensure that your manuscript meets PLOS ONE's style requirements, including those for file naming. The PLOS ONE style templates can be found at
http://www.journals.plos.org/plosone/s/file?id=wjVg/PLOSOne_formatting_sample_main_body.pdf and http://www.journals.plos.org/plosone/s/file?id=ba62/PLOSOne_formatting_sample_title_authors_affiliations.pdf

2. In your Methods section, please provide additional location information of the study areas, including geographic coordinates for the data set if available.

The location information of the study sites was added as supporting information (S1 Table).

3. Please include additional information regarding the survey or questionnaire used in the study and ensure that you have provided sufficient details that others could replicate the analyses. For instance, if you developed a questionnaire as part of this study and it is not under a copyright more restrictive than CC-BY, please include a copy, in both the original language and English, as Supporting Information.

The questionnaire, in both Spanish and English, was added as supporting information (S2 Table).

4. Thank you for stating the following in the Financial Disclosure section:

Funding for this research was provided by the Albert-Heim-Stiftung Project Nr. 132 – (http://www.albert-heim-stiftung.ch, awarded to SD), the Spezialisierungskommission of Bern University (SpezKo) (https://www.vetsuisse.unibe.ch/weiterbildung/spezialisierungskommission/index_ger.html, awarded to FMS), the Wolfermann-Nägeli-Stiftung Nr. 2018/28 (awarded to SD) and the Swiss Programme for Research on Global Issues for Development (r4d programme), Project Nr. 160919, (http://p3.snf.ch/project-160919). The two UAV used during the study were provided by RTI International and the Universidad del Valle.
The funders had no role in study design, data collection and analysis, decision to publish, or preparation of the manuscript.

We note that one or more of the authors are employed by a commercial company: RTI International and/or Remote Intelligence, LLC,

RTI International is not commercial company, it is an independent non-profit research institute.

Remote Intelligence, LLC did not provide any fund for the study and did not provide financial support in the form of author salary or research material. Merlin Benner's salary was provided by RTI International for this study.

1. Please provide an amended Funding Statement declaring this commercial affiliation, as well as a statement regarding the Role of Funders in your study. If the funding organization did not play a role in the study design, data collection and analysis, decision to publish, or preparation of the manuscript and only provided financial support in the form of authors' salaries and/or research materials, please review your statements relating to the author contributions, and ensure you have specifically and accurately indicated the role(s) that these authors had in your study. You can update author roles in the Author Contributions section of the online submission form.

Please also include the following statement within your amended Funding Statement.

“The funder provided support in the form of salaries for authors [insert relevant initials], but did not have any additional role in the study design, data collection and analysis, decision to publish, or preparation of the manuscript. The specific roles of these authors are articulated in the ‘author contributions’ section.”

If your commercial affiliation did play a role in your study, please state and explain this role within your updated Funding Statement.

Funding for this research was provided by the Albert-Heim-Stiftung Project Nr. 132 – (http://www.albert-heim-stiftung.ch, awarded to SD), the Spezialisierungskommission of Bern University (SpezKo) (https://www.vetsuisse.unibe.ch/weiterbildung/spezialisierungskommission/index_ger.html, awarded to FMS), the Wolfermann-Nägeli-Stiftung Nr. 2018/28 (awarded to SD), the Swiss Programme for Research on Global Issues for Development (r4d programme), Project Nr. 160919, (<http://p3.snf.ch/project-160919>) and Vontobel Stiftung, grant 27.04.2017 (awarded to MBG). The two UAV used during the study were provided by RTI International and the Universidad del Valle.
RTI International provided support in the form of salaries for author MB, but did not have any additional role in the study design, data collection and analysis, decision to publish, or preparation of the manuscript. The specific roles of this author is articulated in the ‘author contributions’ section.

2. Please also provide an updated Competing Interests Statement declaring this commercial affiliation along with any other relevant declarations relating to employment, consultancy, patents, products in development, or marketed products, etc.

Within your Competing Interests Statement, please confirm that this commercial affiliation does not alter your adherence to all PLOS ONE policies on sharing data and materials by including the following statement: "This does not alter our adherence to PLOS ONE policies on sharing data and materials.” (as detailed online in our guide for authors <http://journals.plos.org/plosone/s/competing-interests>) . If this adherence statement is not accurate and there are restrictions on sharing of data and/or materials, please state these. Please note that we cannot proceed with consideration of your article until this information has been declared.

Please include both an updated Funding Statement and Competing Interests Statement in your cover letter. We will change the online submission form on your behalf.

Please know it is PLOS ONE policy for corresponding authors to declare, on behalf of all authors, all potential competing interests for the purposes of transparency. PLOS defines a competing interest as anything that interferes with, or could reasonably be perceived as interfering with, the full and objective presentation, peer review, editorial decision-making, or publication of research or non-research articles submitted to one of the journals. Competing interests can be financial or non-financial, professional, or personal. Competing interests can arise in relationship to an organization or another person. Please follow this link to our website for more details on competing interests: <http://journals.plos.org/plosone/s/competing-interests>

Merlin Benner is an owner and employee of Remote Intelligence, LLC, a private, for-profit company, which provide Unmanned Aerial Drone sales and consulting services.  This commercial affiliation does not alter our adherence to all PLOS ONE policies on sharing data and materials.

5. We note that you have indicated that data from this study are available upon request. PLOS only allows data to be available upon request if there are legal or ethical restrictions on sharing data publicly. For more information on unacceptable data access restrictions, please see http://journals.plos.org/plosone/s/data-availability#loc-unacceptable-data-access-restrictions.

In your revised cover letter, please address the following prompts:

a) If there are ethical or legal restrictions on sharing a de-identified data set, please explain them in detail (e.g., data contain potentially sensitive information, data are owned by a third-party organization, etc.) and who has imposed them (e.g., an ethics committee). Please also provide contact information for a data access committee, ethics committee, or other institutional body to which data requests may be sent.

b) If there are no restrictions, please upload the minimal anonymized data set necessary to replicate your study findings as either Supporting Information files or to a stable, public repository and provide us with the relevant URLs, DOIs, or accession numbers. For a list of acceptable repositories, please see http://journals.plos.org/plosone/s/data-availability#loc-recommended-repositories.

We will update your Data Availability statement on your behalf to reflect the information you provide.

All data collected in Guatemala is bound to the data protection bylaws and confidentiality agreements requested by the Ethics Committee of the Center for Health Studies (CES-UVG) of the Universidad del Valle de Guatemala. Given special permission had to be requested to Municipal authorities in Poptún and to two indigenous Maya Q’eqchi’ Councils due to potentially sensitive information, access to further data can be granted upon request following strict guidelines. For such a request please contact the CES-UVG Ethics Committee president, Dr. Jorge Jara at [jjara@ces.uvg.edu.gt](mailto:jjara@ces.uvg.edu.gt) or phone +502 2364 0492.

Responses to the review comments

Reviewer #1:

At the outset, I would congratulate the authors for choosing a very challenging subject and appreciate the rigorous field work untaken.

Thank you for this nice comment.
The manuscript compares the population size estimates of dogs obtained through three methods, (a) a direct count through aerial photographic count from four survey sessions (b) Bayesian statistical model using data from capture –recapture techniques and priors from household interviews, and (c) through human: dog population ratios estimation through information collected through interviews of selected sample of residents, with the owned dog population estimate obtained through census.
While the procedure of the methods used have been described well, the very purpose of designing such a study needs to be brought out emphatically, which I feel is lacking as the manuscript stands now. In other words, why would one attempt UAV aerial counting? Are the methods previously used are inadequate? If yes, how will this method mitigate? I would suggest also discussing the economics of using such methodology over other methods.

Thanks for pointing this out. It has been addressed lines 104-109.

Also, the title of the manuscript includes only one aspect (i.e. use of UAV) and precludes other methods used or the important aspect that the manuscript compares the different methods.

The title of the publication has been adapted.

The detailed comments are as follows:

Line 73-75: Although, underestimation could be an issue with some of the methods, it is not understood how capture-recapture technique precludes the ownerless free-roaming dogs. It will be good to elaborate and also cite the work where this is the case.

Following the comments of a reviewer 2, this sentence has been removed because misleading. Many capture-recapture methods account for detection heterogeneity in wildlife. We aimed to say that methods accounting for heterogeneity in detection probability have not been widely used with FRDD.

Line 77-78: It will be good if you could provide examples of priors that have been used earlier with references, please.

The methods used in this study to estimate the values of the prior are the same than the ones used in Kayali's, Dürr's and Gsell's publications. This has been indicated in the methods part lines 357-359.

Lines 106-110: The objective of the study aligns with the title, however, when we look at the results a basic query that comes up id that if using UAV is not helpful and if we still have to compare it to census (I am not sure if you consider it the gold standard), why use it at all! It is in this context that I suggest that the Bayesian method and human : dog ratio method should be included in the aim as well (and also in the title).

We agree with this point. The title has been adjusted. We also adjusted the objectives of the study to not focus on the UAV only, but to discuss different methods to perform dog population size estimations with their benefits and drawbacks (line 113-116).

Line 129: Please provide the date the data was accessed

The date has been added to the text line 136.

Line 145: Please provide the source.

These data have not been published yet (indicated at line 160).

Line 147-151: How can one differentiate between free-roaming and confined dog from the aerial pictures (also the how can one differentiate between owned confined and owned free-roaming dog from the serial pictures)? Suggest provide an explanation here.

It is impossible to differentiate confined and free-roaming dogs on the aerial pictures, that why we consider them together in the UAV method. This is specified lines 449-450 and line 617.

Line 202-203: Why was morning and late afternoon time chosen?

This is when free-roaming dogs might have peaks in activity. Comment addressed line 265-267.

Table 2: Why does the flight time vary considerably? There is a wide time difference between the flight times within similar temporal sessions. However, the difference in flight times is not associated with the number of pictures taken. Can you elaborate on this?

The reason for this difference is most probably the light condition. The system adjusts flight speed depending on ambient light conditions to avoid motion blur. Explanation added to Table 2's caption lines 282-284.

Line 233-240: Suggest provide a flow chart for easy understanding.

A flowchart was added, as a figure, line 308 in the manuscript.

Line 272: ‘Cultural beliefs’ such as?

Cultural beliefs related to blood drawing and injections described lines 233-236.

Line 273: How did you know a dog was four months? (please mention it was based on visual appreciation, if it was so).

By asking the owner. Comment addressed line 282.

Line 277-286: Were the interviews conducted on all dog owning households? Or on selected ones? If on selected ones, how was the sampling done? Why were the non-dog owning residents not included? If only dog owners were included would it not introduce a bias in the ’prior’ estimation?

The interviews were conducted with all dog owning household whose owner was present and accepted to participate in the study. They are the owners of the collared dogs.

They were chosen because only dog owning resident could give information about dog keeping practices, such as confinement used as prior information.

We assumed that dog owner would be more aware of the dog population structure than the non-dog owning resident, who could have assumed that many dogs were ownerless because they do not know the dog nor the owner. The absence an ownerless dog population also fits the information given by the researchers from "One Health Poptun" who followed La Romana and Sabaneta communities during three years. This is discussed lines 656-665.

Line 294: What about the dogs in the buffer zone?

The model has been adapted to take into account the percentage of the area that has been covered during the foot-patrol transects (also based on the comments of reviewer 2). The dog population size estimate has been adjusted accordingly.

Line 309: Do you mean ‘lose’?

Some dogs were found without collar when we removed the collars. They might have lost the collar by themselves or a person might have removed it. The owners generally did not know what happened. This has been clarified at line 364.

Line 314: What is the basis of 10% assumption? (In other words what is the likely dog population size for this assumption of 10%)?

We have adapted the Bayesian statistical model and setting of such a lower limit it not further needed.

Line 398-400: Probably better suited to be part of discussion section.

We agree and deleted this sentence from the results. It has already been stated in other words in the discussion (line 626-628).

Lines 401-403; 406-408 & Table 5: Suggest round-off the number of dogs to whole numbers.

As suggested, the we rounded-off the numbers lines 474-475, 479-480, 486, 524-531. The model was modified as suggested by one of the reviewers and some of the estimates have changed.

Table 2, 4 & 5: please give the first row/column heading.

It has been done for the three tables

Line 436: Did you mean the owned pups? In that case what about the ownerless pups?

The ownerless pups are probably not detected by any of the methods for the same reasons that the owned pups but because we do not have any information distinguishing between ownerless adults and pups, we consider the ownerless dog population as a whole. This should have no impact on the estimate since they are no ownerless dogs in the study areas. Comment added line 505-506.

Line: Please say how the dog census was done, and when.

The dog census has now been described in the methods, line 152-161.

Line 448- 452: Please reword to provide clarity.

This sentence has been removed because we do not compare the number of dogs detected with the UAV and the estimates of the capture-recapture model anymore. We now compare the number the dogs spotted during the UAV and the foot-patrol transects lines 451-452.

Line 450: Which two methods? Please explain for the benefit of the reader.

This was the UAV numbers and the transect walks. However, we do not anymore compare these two methods directly and therefore deleted this sentence.

Line 476-477: “fully open environment”. Is it practically possible to drive all free-roaming dogs into an open environment to count them? I am not sure about it.

The recommendation is not to drive free-roaming dogs to a specific open area but to select the method based on the type of area, where the study takes place. We recommend to use the UAV method only in areas which are already open because the method do not fit in areas covered by trees for example. In fully covered areas, other methods have to be used. The discussion part for the UAV method has substantially revised and this sentence was reformulated lines 575-576.

Secondly, I think you already used the temporal settings as the ones you are suggesting (morning and late afternoon), but it yielded low counts!

As well, this sentence has been removed.

Line 486: Suggest explain briefly about ‘deep learning’ the references are great, but a brief context here will help the reader).

'Deep learning' is now explained lines 590-593.

Line 481: Infrared thermal imagery methods: how would they preclude other animals in rural areas such as calfs/foals/sheep/goat etc.? Also will it be economically viable?

These comments have been addressed lines 598-606.

Line 506: If wolves can be confused for rocks, the vice versa could also be true, and we can miss some dogs presuming they are rocks!

This is true. The confusion can occur in both directions. Line 568-569.

Please also discuss the advantages of the use of UAV in dog population estimation supported by references.
As suggested, the advantages of using UAV in FRDD population size estimation have been developed lines 548-554.

## Reviewer #2:

The authors used UAVs, foot-patrol transect surveys, and human:dog ratios to attempt to estimate free-roaming dog population sizes for three separate study areas in Guatemala. Although I agree that the scientific foundation for attempting to make such comparisons is generally solid, I have a few major criticisms of the authors’ approach to arriving at those ph, based entirely on the survey designs and statistical analyses.

Thank you for the very relevant comments and suggestions. We tried to address them as well as possible with our available data.

1. The data collected from the UAV surveys were not analyzed. The authors treat these data solely as raw counts of the number of dogs visible in the photographs obtained via the UAVs. At best, these results are simply indices, not estimates, of the minimum population sizes of dogs that are detectable via aerial surveys. As the authors note on lines 233-240, 384-388, and 464-468, these UAV data are imperfect detection data with a large proportion of dogs being undetected. The reasons for this are numerous, some of which the authors acknowledged (poor quality photos, vegetation cover, flight altitude, etc.), whereas others were not considered (e.g., distance and angle of dog from UAV transect when photo was taken). Yet, none of those factors were included in any formal analytical framework. To be clear, the UAV transect surveys are analogous to airplane or helicopter transect surveys, which constitute distance sampling with imperfect detection. Thus, the authors need to analyze the UAV data collected from Sabaneta and Poptun using a model that accounts for the distances between the UAV transect and the locations of detected dogs, as well as imperfect detection. Given many of the dogs in the study area were marked and had known identity, these data represent mark-resight distance sampling data, which the modified MRDS models developed by Borchers et al. (2015; J. Am. Stat. Assoc. 110:195-204) are well suited to analyzing. Alternatively, because the spatial locations of each UAV transect and detected dog are available, the authors could discretize the survey area as a gridded or transect state space (Russell et al. [2012] J. Wildlife Manag. 76:1551-1561; Thompson et al. [2012] J. Wildlife Manag. 76:863-871; Morin et al. [2016] 80:824-836; Murphy et al. [2018] Ecosphere 9:e02479) and apply a spatially explicit mark-resight model to estimate population size (Chandler and Royle [2013] Annals Appl. Stat. 7:936-954; Sollmann et al. [2013] Ecology 94:553-559; Murphy et al. [2019] Sci. Reports 9:4590).
We completely agree with this comment and thank the reviewer for pointing this out. Unfortunately, the collars were not visible on the UAV picture, which prevented the utilization of CR or SCR models mentioned above. In addition, we lacked information on how environmental factors (distance with the owner's household, land use) impact the FRDD roaming behavior and could not develop a model giving reliable estimates. We completely reshaped the discussion of the UAV picture analysis and also clearly state that the counts of the dogs in the pictures are raw data and no estimates of the dog population. Lines 495-497, 608-632.

2. The foot-patrol transect surveys also represent distance sampling with imperfect detection. However, the authors’ model does not accommodate any distance measurements and therefore heterogeneity in detection due to spatial proximity between the transects and dogs is unmodeled, leading to estimates that are likely biased to an unknown magnitude. These data are also mark-resight distance sampling, given detections of both marked and unmarked dogs were recorded at varying distances from the walked transects. Thus, similar to above for UAV transects, an MRDS or spatial mark-resight model should be used to analyze these detection data.
Unfortunately, we do not have data on the distance and angle between the observers and the spotted dogs, nor their GPS location. However, we adapted the model and included a recapture probability that takes into account the coverage of the foot-patrol transect survey. We calculated a minimum and a maximal coverage value to account for the fact that some of the dogs might not be visible because of the distance from the transect line. It would be better to include in the model individual distance measurements. The methods and discussion part for this method has therefore been largely modified according to the new model used lines 373-419 and 684-693.

3. “Bayesian statistical model” is an extraordinarily vague term that does not accurately represent the model that the authors used to analyze the foot-patrol transect survey data. The authors must be specific about this model throughout the manuscript: This is a non-spatial mark-resight model, with some minor modifications for mark loss and ratio of owned:ownerless dogs, which was fit in a Bayesian inferential framework. Mark-resight models that accommodate mark loss have been available in the frequentist inferential framework for a decade (e.g., McClintock et al. [2009] Biometrics 65:237-246; McClintock [2018] Chapter 18 in Cooch & White (eds.), Program MARK: A Gentle Introduction). Thus, the fact that the authors fit their model in a Bayesian framework is relatively unimportant. Furthermore, as noted above, the specification of the authors’ model was insufficient for accurately estimating dog population size from the foot-patrol transect data. At minimum, a model needs to be used that accounts for spatial distances between the transects and detected dogs; evaluates variation in detection probability due to survey, environmental, and/or individual covariates; and does not assume that both marked and unmarked dogs have the same spatial distributions relative to the marking/transect locations.

The name of the model used have been adapted throughout the manuscript and is now called CR model (for capture-recapture). Also, the model has been modified to include a spatial component, that is the area under observation, included in the coverage probability (new Table 3 and described in the methods, line xxx-xxx). Due to the new model, the estimates of the dog population size via this method changed slightly (Table 6), but the interpretation stays the same. However, we did not have data on variation in detection probability due to environmental and individual covariates, nor on marked and unmarked dogs spatial distributions. We therefore did not include these components into the CR model. This is discussed lines 373-419 and 621-632.

Minor Comments:
Data: The detection data derived from the photographs, not the photographs themselves, need to be provided. The results are not reproducible without the detection data.

The detection table has been added to the supporting information (S3 Table).

Lines 4-6: This title is misleading. It suggests that UAVs were used to estimate dog population size, but they were not. The UAV surveys only provide a count of minimum number of dogs, because the data were not analyzed using any modeling approach.

The title has been adapted according to your comment.

Lines 73-75: These statements are not true. Capture-recapture and mark-resight models that account for such heterogeneity via either finite mixtures (Pledger [2000] Biometrics 56:434-442; Gardner et al. [2010] J. Wildlife Manag. 74:318-325) or individual covariates (Huggins [1989] Biometrika 76:133-140) have been around for decades.

We apologize for the misunderstanding. We meant that models accounting for such heterogeneity have not been widely used in FRDD population size estimation only. The sentences was very confusing and has been removed.

Lines 76-78: As noted above, the term “Bayesian statistical models” is too vague for use here. Bayesian is just an inferential framework and all models are statistical models. Additionally, as noted above, frequentist models are also able to account for heterogeneity in the population.
Line 356: Again, this is a capture-recapture (mark-resight) model that was fit in a Bayesian framework, NOT a “Bayesian statistical model based on capture-recapture data”.

This has been adapted. Please refer to the responses in the comments above.

Lines 264-265: Why were the location data recorded by these GPS collars not incorporated in a spatially explicit model? Doing so would improve accuracy of the population size estimates (Sollmann et al. 2013, Murphy et al. 2019).

This was not possible because the GPS number was not visible from meters away. Even if a spotted dog had a collar, we could not individually identify them, as the study was not designed for this purpose. Therefore, we couldn't retrieve information about its GPS position, at the time of the transects.

Lines 292-300: This describes mark-resight distance sampling data, but the authors’ model is not a mark-resight distance sampling model.

The model has been adapted, please refer to the comments above.

Lines 384-388: Then why was heterogeneity in detection due to these factors not included in a model to formally analyze the UAV data?

This was investigated but because we do not have information on how these factors impact dog roaming behavior, we could not obtain reliable estimates. Discussed lines 621-632.

Lines 419-420: Misleading statement. The UAV data were not used to estimate population size, only to produce a minimum count.

We agree, the sentence has been removed.

Lines 464-474: Then a model needs to be used that incorporates heterogeneity in detection.

This is discussed lines 608-632.

Lines 493-498: Why was this not done? This is a major weakness of the paper and study.

Unfortunately, CR could not be used because the picture quality was too low to assess if a dog was marked or not. This is addressed lines 448-449, 610-613.

Lines 533-544: Capture-recapture models fit in a frequentist framework can also do this, so this statement seems irrelevant.
We agree, the sentence has been removed.

## Reviewer #3:

I revised the manuscript "Using Unmanned Aerial Vehicles (UAV) for free-roaming dog population size estimation" by Warembourg et al.
The topic of this study is of interest and the results are novel and useful. The unprecedented use of drones to estimate dog abundance is relevant.
On the other hand, some points need to be properly adressed to improve the quality of the manuscript.

Thanks for the comments and suggestions.

-The abstract must present the values of population size estimates. This will be useful for a better understanding of the poor sensitivity of UAV.

Estimates are presented in the abstract lines 37-45.

-I believe that the inclusion of pictures and/or photos of study areas would be important for a better understanding of the characteristics of the regions, especially to an international readership.

As suggested, pictures of the study area have been added to the manuscript. Lines 149, 310, 328, 354.

-The"One Health Poptun" research and methods of domestic animal census should be better presented.

The dog census method has been detailed lines 152-161.

-The small number of flights should also be pointed as a factor that limited the sensitivity of UAV. Authors must justify why they have only conducted 11 flights.

Following the comments of another reviewer, we decided to compare the number of dogs counted during the UAV transects to the number of dogs counted during the foot-patrol transects and not to the dog census anymore. The number of flights is therefore not a limitation because we flew at least three times in each study areas and it is likely that the maximum number of dogs spotted per flight would stay low if we flew the UAV again under the same flight parameters.

-I understand the ethical issues that prevent authors from providing all drone images. However, some pictures should be inserted in the article to exemplify information presented. This is possible and does not violate ethical standards.
Suggestions.
--->"Pictures that could not be analysed due to poor quality"
--->Pictures in which dogs could be identified.
--->Pictures that generated doubts.
--->Pictures mentioned in lines 243-245 ("doubtful observations resembling dogs but not presenting dog characteristics, such as ears, thorax, pelvis, limbs or tails, were not counted as dogs").
---> Pictures demonstrating the effect of climatic conditions on identification.
--->Photographs made during transect walks.
As suggested, pictures have been added to the manuscript.

-Concerning the information presented in lines "243-245": "doubtful observations resembling dogs but not presenting dog characteristics, such as ears, thorax, pelvis, limbs or tails, were not counted as dogs".
Why did the authors adopt this criterion?

This is explained lines 320-322.

What are the implications for the sensitivity of the technique?

This is discussed lines 569-571.

It is highly recommended that further analysis with an alternative criterion be conducted by the authors.

Thank you for your recommendation. Because of the UAV picture quality, it is difficult to use an alternative criterion. Therefore, instead of further analyze our pictures, we would recommend for further research to improve the quality of the data collected by flying at a lower altitude first. This is discussed in the section about the use of the UAV.

-The effects of other study limitations should be further discussed:
-->Non-collared dogs (lines 269-271)

This is should not be an issue for the population size estimation because CR model is based on the assumption that not all the dogs can be collared.
-->Refusals (lines 271-273).

This is discussed lines 535-543 for the dog census survey and lines 662-664 for the foot-patrol survey.

-With regard to the figures
--->Figure 3 should be better explained in the caption.
--->I did not understand the figure 4.
--->I believe that figure 5 should be inserted in the methodology.

Figure 3 and 4 have been exchanged by mistake during the submission process and did not fit the captions. I hope this new version is clearer.

Figure 5 has been removed because we do not compare the UAV raw counts and the estimates of the CR model anymore.

-Authors should check all links, as some pages did not open.

Thank you for pointing this out. This has now been corrected.
