# Supplementary material for: Estimation of free-roaming domestic dog population size: Investigation of three methods including an Unmanned Aerial Vehicle (UAV) based approach
Source: PLoS One. 2020 Apr 8;15(4):e0225022. doi: 10.1371/journal.pone.0225022 (PMC7141685; doi:10.1371/journal.pone.0225022)
Supplement: Attachment — Submitted filename: Reponse to Reviewers.docx [file pone.0225022.s012.docx]

6. Review Comments to the Author

Please use the space provided to explain your answers to the questions above. You may also include additional comments for the author, including concerns about dual publication, research ethics, or publication ethics. (Please upload your review as an attachment if it exceeds 20,000 characters)

Reviewer #1: The article will immensely contribute towards future research on dog population management. Recommended for acceptance.

Many thanks for your comments and recommendation. We are pleased that the revised manuscript fit your expectations.

Reviewer #2: I applaud the authors for making quality revisions to their manuscript and addressing our previous comments. As a result of their efforts, I think the manuscript is very much improved. I do have some lingering minor comments for the authors to consider:

Many thanks for your review, it was very helpful in improving the quality of the manuscript. We hope our revised version of the manuscript will fix the last remaining points.

1) Lines 226-227: I am still concerned about the choice to not use the GPS locations from the marked dogs, whose collars had a GPS chip, in the analyses. The locations obtained from those GPS chips could help the authors determine which dogs in the photos from UAV survey were marked versus unmarked. With 71 and 96 dog detections via the UAV flights at Poptun and Sabaneta, respectively, sufficient data exist to estimate the population size for both of those locales using a mark-resight model if the locations from marked dogs' GPS collars are used to differentiate between dogs in photos by their marked vs unmarked status. A similar approach is used when sampling via camera-traps and some animals in a population have GPS collars (e.g., see Whittington et al. [2018]: <https://besjournals.onlinelibrary.wiley.com/doi/full/10.1111/1365-2664.12954>; Murphy et al. [2019]: <https://www.nature.com/articles/s41598-019-40926-7>, among others). I see no reason why this cannot be applied to the UAV data to discriminate between marked and unmarked dogs, which would allow the authors to produce population size estimates from UAV flights for two of the three sampled locales.

We agree that it would be a good idea. However, the GPS data were meant to be used for another study and not to determine which dogs were marked during the UAV transect survey. Therefore, the GPS settings have not been optimized for this purpose. Because of the limited battery capacity, many GPS stopped recording before or during the transect survey. The percentage of GPS collars recording during the first day of the UAV transect survey ranged from 57% to 70% of the collared dogs and decreased during the second day (38% to 54% of the dogs collared). In addition, the accuracy of the GPS was low when the dog was inside a house, which might artificially locate dogs on the streets or yards when a dog is actually inside a building. This is an issue for the small scale settings we are dealing with in our situation of estimating dog population size in a domesticated area. Because of all these reasons, it is impossible in our study to use the GPS data and it might also be difficult in future studies. Dogs might spent time inside their owner's houses even if they are completely free to roam, which makes their GPS locations difficulty reliable for short distances.

2) Table 6: The caption indicates that estimates from the UAV survey are in included, but they are not. Also, why are the values for mean number of ownerless dogs all zeros?

Thank you for pointing it out, the UAV survey has been removed from the caption. Concerning the zeros, the numbers were rounded to integer (as recommended in the first review round) but we finally decided to keep two decimals to avoid any confusion.

3) Lines 512-519: This information also needs to be presented upfront in the Methods section for each approach. Also, if the CR model does not "consider confined dogs" (line 514), then why is a confinement probability included in the model?

The CR model consider partially confined dogs but not completely confined dogs. This sentence has been adapted line 514. The fact that we consider confinement and ownership in the model was already presented in the methods part (line 365-371). However, we would like to keep the discussion on Fig 9 in the discussion part only.

4) Lines 577-578: This is a very questionable recommendation. If researchers follow this, then considerable sampling bias will be induced in the estimates.

The sentence has been adapted lines 577-580.

5) Lines 592-608: Way too much discussion and space devoted to something that the authors did not use. This stuff about deep learning needs to be reduced to 2-3 sentences maximum.

This part has been expanded as a result of another reviewer's comment and we therefore would like to keep this part in the discussion.

6) Line 610: But earlier in the Methods and Discussion, the authors stated that confined dogs can be detected by UAVs. Which is it?

Dogs confined in yards are visible on the UAV pictures, dogs confined inside a house are not visible. This has been clarified 611-612.

7) Lines 614-615: Deploying GPS collars on dogs would probably be a more fruitful approach, because the locations from each marked dog can be used to determine where and when a marked dog was located relative to the UAV flight time and flight path.

For domestic dog population size estimation, we do not think that the use of GPS collars is a valuable method, because of reasons pointed out in our response to comment 1. In addition, even if we can overcome these limitations, it would not be cost-effective for policy makers to use a method needing both drones and GPS collars to estimate the dog population size. We therefore decided to not modify the text at these lines of the manuscript.

8) Lines 623-634: All of these are not restricted to spatial models, but apply to non-spatial models as well, including the authors' model.

This is right and it has been corrected line 625.

9) Lines 658-662: These are very strong assumptions that may not represent reality.

A sentence has been added lines 659 and 666-667.

10) Lines 675-679: Unclear why this would not be critical if the aim is to estimate FRDD population size, given a 5.8% influence is not negligible.

We agree that 5.8% change of dog population size might not be negligible. We therefore deleted a sentence saying that it is not a critical parameter. Sentence adapted lines 679-683.

11) Lines 686-692: The authors need to provide relevant citations for these statements.

Thanks for pointing it out. References have been added to the manuscript line 697.
